# Age at first birth, age at menopause, and risk of ovarian cyst: a two-sample Mendelian randomization study

**DOI:** 10.3389/fendo.2023.1279493

**Published:** 2024-01-04

**Authors:** Qian Su, Zhiyong Yang

**Affiliations:** Clinical Medical College and Affiliated Hospital of Chengdu University, Chengdu University, Chengdu, China

**Keywords:** Mendelian randomization, ovarian cyst, age at first birth, age at natural menopause, genetic epidemiology

## Abstract

**Background:**

Increasing observational studies have indicated that hormonal reproductive factors were associated with ovarian cyst, a common gynecological disease. A two-sample Mendelian randomization (MR) was carried out by investigating the causality of reproductive factors including age at first birth (AFB), age at natural menopause (ANM), and age at menarche (AAM), and the risk of ovarian cyst (OC).

**Method:**

Summary statistics were collected from a large genome-wide association study (GWAS), and we used a two-sample MR study to clarify the causal association between the exposure of AFB (*N* = 542,901), ANM (*N* = 69,360), and AAM (*N* = 29,346) and the outcome of the OC (*N*_case_ = 20,750, *N*_control_ = 107,564). We separately selected 51, 35, and 6 single-nucleotide polymorphisms (SNPs) as instrumental variables (IVs) for assaying the influence of AFB, ANM, and AAM on OC, respectively. Then, the causal relationship was tested through multiple approaches including an inverse-variance weighted method, an MR-Egger regression, and a weighted median method. In addition, the MR-PRESSO method was also used to verify the horizontal pleiotropy. Subsequently, we adjust the confounders for MR design.

**Results:**

The MR analysis results showed that AFB was negatively associated with the OC (IVW Beta: −0.09, OR: 0.91, 95% CI: 0.86–0.96, *p* = 0.00185), and the greater AAM decreased the risk of OC (IVW Beta: −0.10, OR: 0.91, 95% CI: 0.82–0.99, *p* = 0.0376). However, ANM has a positive correlation with the OC (IVW Beta: 0.05, OR: 1.05, 95% CI: 1.03–1.08, *p* = 8.38 × 10^−6^). After adjusting BMI, alcohol intake frequency, and ever smoked, we also obtained a negative relationship between AFB and OC (*p* < 0.005). Meanwhile, we adjusted weight, alcohol intake frequency, and height, and then found a causal relationship between older AMN and an increased risk of OC (*p* < 0.005).

**Conclusion:**

A causal effect of reproductive factors on the development of OC, affected by AFB, ANM, and AAM, was found convincingly. After adjusting the confounders, we also successfully found the substantial causal effect of younger AFB, younger AAM, and older ANM on an increased risk of OC.

## 1 Introduction

Ovarian cysts (OCs) showed a high incidence of 21.2% among healthy postmenopausal women in Europe, which affect approximately 7% of women at some point around the world (1). As a result of ovulation, a fluid-filled sac known as an OC can form on one or both ovaries. It is not common to find adnexal masses or OCs in women, and approximately 20% of women developed at least one pelvic mass in their lifetime (2). The sample OC could be found by ultrasound, and the sample OC was fairly common and appear stable in a majority of postmenopausal women with no intervention measure (1). Nevertheless, multiple complications such as blood loss, cyst rupture, and pelvic pain could occur during the development of OC (2). In early studies, researchers found that a greater age at first birth (AFB) was the main risk factor associated with serous or mucinous OCs (3). Observational research identified the association between hormonal reproductive factors (such as AFB) and OC (4). Therefore, it will be of great use to test the causal effect between hormonal reproductive factors and OC.

AFB poses a substantial impact on health and evolutionary fitness. Pregnancy is an important factor that affected the future health status of women. Significant alterations in endocrine hormone profiles, endocrine gland morphology on imaging, and serum and urine electrolytes may occur due to the physiological changes in pregnancy (5). During pregnancy, estradiol levels, levels of progesterone and 17-hydroxyprogesterone, and testosterone levels progressively rise, while follicle-stimulating hormone and luteinizing hormone (LH) levels are low (5). Therefore, the changes in hormone level may affect the formation of OC. In addition, several studies have investigated the reproductive factors of AFB and the risk of disease. For example, Luo’s group found that older AFB is associated with an increased risk of pancreatic cancer in women through a meta-analysis (6). Yang’s group analyzed NHANES data, providing evidence that women with younger AFB have higher odds of non-alcoholic fatty liver disease in later life (7). Li’s group carried out a meta-analysis to evaluate the melanoma risk correlated with AFB (8). However, researchers hardly focused on the AFB and the risk of OC.

Age at natural menopause (ANM) has implications for women’s quality of life and health. Menopause causes changes in hormone levels, leading to some impact on women's lives. Specifically, with the decreasing estrogen, women have a higher risk of suffering from some illness such as osteoporosis. On the basis of the related research, during menopause transition, some sex hormones showed large fluctuations, such as sex hormone-binding globulin (SHBG) and bio-available testosterone, which may also be involved in menopause-related diseases (9). Later ANM was associated with higher bone mineral density, a longer life expectancy and lower risk of fracture, cardiovascular disease (CVD), all-cause mortality, and cardiovascular death, yet with greater breast and ovarian cancer risk (10). The increasing evidence supporting age at menopause onset as a marker of overall health calls for worldwide attention. Michael’s cohort study included 144,260 postmenopausal women and found that menopause (before age 40 years) increased the risk for cardiovascular diseases (11). A Mendelian randomization (MR) also found the causal effect relationship between ANM and the risk of breast cancer (12). However, the real relationship between ANM and OC remains unclear.

Here, we conducted an MR to provide a reliable estimation between the reproductive factors (AFB, ANM, and AAM) and an outcome of OC. An MR study applied single-nucleotide polymorphisms (SNPs) as instrumental variables (IVs), which were from the genome-wide association study (GWAS) (13, 14). IV–exposure associations were extracted from the largest GWAS(s) accomplished in AFB (*N* = 542,901), AMN (*N* = 69,360), and AAM (*N* = 29,346) (15, 16), and the data source is shown in **Supplementary Table S1**. IV–outcome associations were extracted from a large GWAS accomplished in OC (*N*_OC_ = 20,750, *N*_control_ = 107,564).

## 2 Methods

### 2.1 Study design

**Figure 1:**
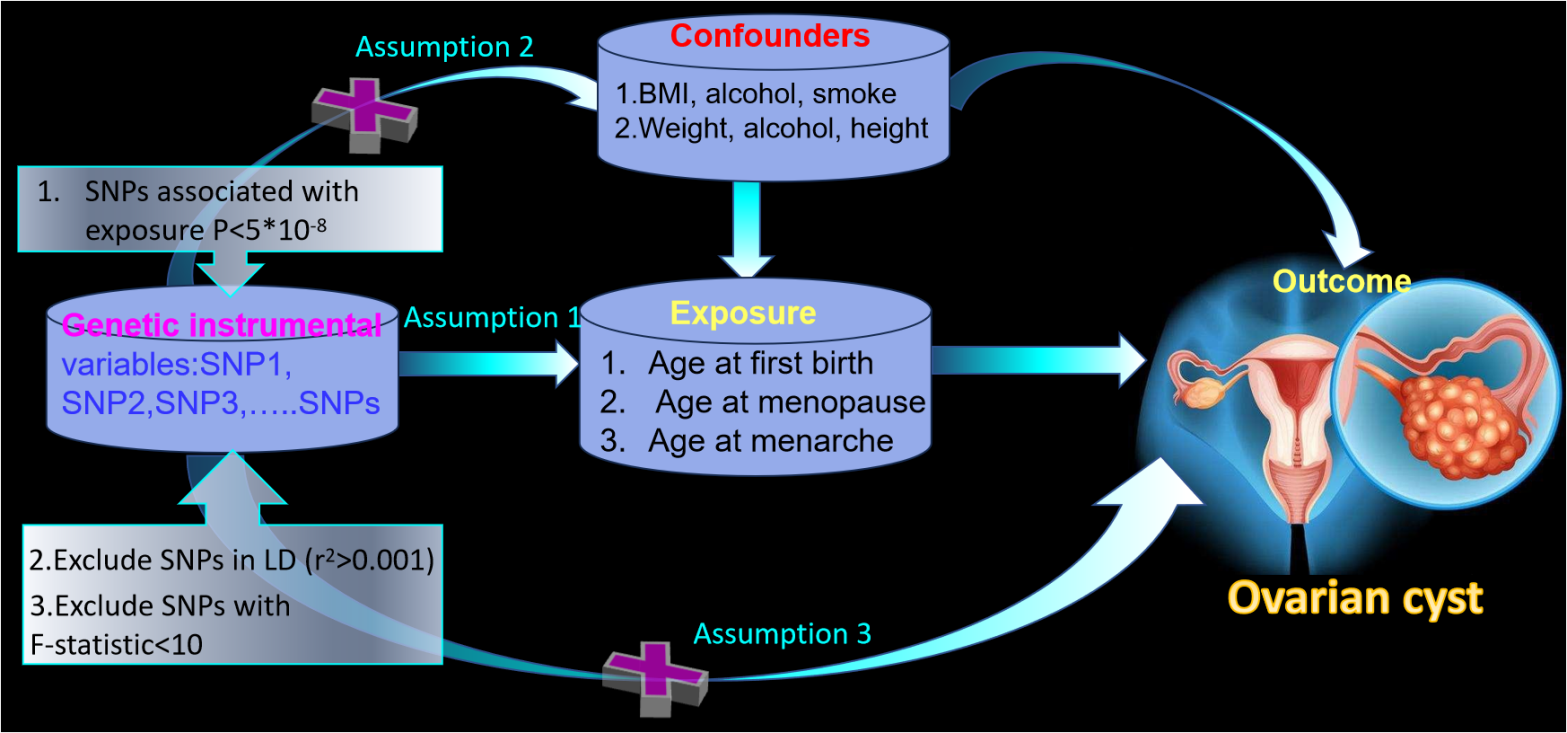
The framework of the two-sample Mendelian randomization study between age at first birth, age at menopause, age at menarche, and ovarian cyst.

We carried out the two-sample MR analysis with the flow-process diagrams shown in **Figure 1**. To clarify the MR analysis, we should follow three important assumptions (13). Assumption 1 states that the IVs of SNPs should be strongly related to exposure (*p* < 5 × 10^−8^). Assumption 2 required the IVs to be irrelevant to any confounders and we should remove the SNPs associated with the outcome. Next, the IVs have an impact on the outcome only through the exposure in Assumption 3. Additionally, all SNPs possessed the *F*-statistic larger than 10 to confirm a robust IV. We decreased the population stratification by including only European ancestry.

### 2.2 Equation data sources

#### 2.2.1 GWAS summary statistics of AFB and ANM

The genetic architecture of AFB has been collected by a GWAS of 542,901 individuals (*n* = 124,088 male individuals; *n* = 418,758 female individuals) from 36 studies; the age of AFB individuals ranged from those born before 1941 (0.60) to those born after 1960 (0.31). In addition, the GWAS was restricted to individuals of European ancestry who passed quality control, and the researcher found that polycystic ovarian syndrome may cause AFB later, which is associated with infertility (15). Individuals were eligible for inclusion in analyses if they met the following conditions: (a) have given birth to a child (parous), (b) all relevant covariates (year of birth) were available for the individual, (c) were successful genotypes genome-wide (recommended >95%), (d) passed the cohort-specific standard quality controls, and (e) were of European ancestry. The genetic architecture of ANM was obtained by a GWAS of up to 69,360 women of European ancestry, which identified the enrichment of signals in/near genes involved in delayed puberty and discovered the first molecular links between the onset and the end of reproductive lifespan (16). The research (16) included women with ANM who were 40–60 years of age, excluding those with menopause induced by hysterectomy, bilateral ovariectomy, radiation, or chemotherapy and those using hormone replacement therapy (HRT) before menopause. For AFB, ANM, AAM, BMI, alcohol intake frequency, ever smoked, and height, the GWAS data were from the publicly available IEU Open GWAS Project database (https://gwas.mrcieu.ac.uk/), and the detailed information is shown in **Supplementary Table S1**.

#### 2.2.2 GWAS summary statistic of OC

GWAS summary statistics for OC were obtained from FinnGen consortium R9 release data (17). The GWAS included 128,314 Finnish adult female subjects and consisted of 20,750 cases and 107,564 controls.

### 2.3 Ethical approval

This MR study was conducted by virtue of publicly published studies or shared datasets, and the datasets had obtained ethical approval and informed consent. We did not have to make any additional ethics statement or consent.

### 2.4 Instrumental variable selection

Our independent IV was defined as follows: met the genome-wide significance threshold of *p* < 5 × 10^–8^, all of which was under the limited value (*r* ^2^ < 0.001 within a clumping window of 10,000 kb) in linkage disequilibrium (LD) analysis (18). Our analysis removed the palindromic SNPs, which was regardless of allele frequency (18). In addition, IVs were included only when they existed in the GWAS summary statistics of outcome, and our analysis did not include the proxy SNPs (19, 20). *F*-statistics (*F* = Beta^2^/SE^2^) were used to evaluate the power of each SNP (21). Eventually, all the SNPs were equipped with stronger statistical power (*F*-statistics > 10).

In this MR study, 51 SNPs for AFB and 35 SNPs for ANM were extracted from the GWAS summary statistics with the outcome of OC. The *F*-statistics of the above SNPs were in the range of 532.08–3,770.81, 349.41–8,923.43, and 30.25–119.80, respectively, for AFB, ANM, and AAM, showing the strong validity of the selected SNPs. Detailed information of all selected SNPs of AFB, ANM, and AAM can be found in **Supplementary Tables S1**-**S5**.

### 2.5 Mendelian randomization estimates

Several approaches were utilized to conduct the MR analysis, including an inverse-variance weighted approach (IVW), an MR-Egger regression, a weighted median approach, a weighted mode, and the MR-PRESSO method.

We applied the IVW method as the primary method for two-sample MR tests. The important condition for IVW estimates is that all instrumental variants are valid, while the weak IVs tend to underestimate the true variation (22). MR-Egger, MR-PRESSO, and the weight median were mutually complementary and estimated the horizontal pleiotropy (23). The MR-Egger approach provided a valid estimation of the null causal hypothesis and the causal effect fit well even with the invalid IVs. The weighted median method was identified as more robust to test the horizontal pleiotropy (24). When 50% of genetic variants were considered to be invalid, this method was robust to outliers and gave unanimous estimation (23). MR-PRESSO was used to evaluate the horizontal pleiotropy through three of its components: the global test, the outlier test, and the distortion test (25). Additionally, the weight mode method could test the causal effect of the subset with the largest number of SNPs by clustering the SNPs into subsets resting on the resemblance of causal effects (25).

Subsequently, for assaying the heterogeneity and pleiotropy, we applied leave-one-out sensitivity analysis, the MR-Egger intercept test, and Cochran’s *Q* statistic (24). Then, we removed the confounders to analyze the direct effect of exposure on outcome. As for the causal effect of AFB and OC, we adjusted the BMI, alcohol intake frequency, and ever smoked. Investigating the causal effect of ANM and OC, we adjusted the weight, alcohol intake frequency, and height.

All analyses were performed using the TwoSampleMR (version 0.5.7), Mendelian Randomization (version 0.8.0), and MRPRESSO package (1.0) in R Software 4.3.1 (https://www.R-project.org). In addition, the *p*-value was less than 0.05 for statistical significance.

## 3 Results

### 3.1 Causal association of AFB on OC through two-sample MR

**Figure 2:**
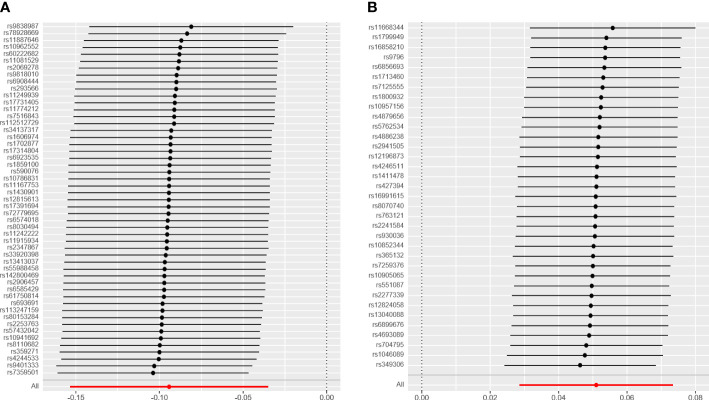
The leave-one-out analysis plot. **(A)** Leave-one-out plot for age at first birth and ovarian cyst. **(B)** Leave-one-out plot for age at menopause and ovarian cyst.

As shown in **Table 1**, there was convincing evidence to support a causal effect between the two hormone-related exposure and the risk of OC. In the main IVW analysis, per-year increase in AFB was associated with 0.09 standard deviation decrease in the risk of OC (OR = 0.91, 95% CI = 0.86–0.96, *p* = 0.00185). The test results kept significant by the other two methods (OR _per-SD increment in AFB_ [95% CI] for weighted median 0.92 [0.85–0.99] and for MR-PRESSO 0.91 [0.86–0.97]). Meanwhile, the MR-Egger regression and weighted mode results were not significant. There seemed to be no apparent sign of pleiotropy (*p*-value of MR-Egger intercept = 0.362). In addition, MR-PRESSO estimation did not observe any outlier SNPs. Next, as depicted in **Figure 2A**, none of the SNPs crossed the zero line in the leave-one-out sensitivity analysis, which proved the non-heterogeneity of our study. Additionally, the MR-PRESSO global test has a *p*-value of 0.057, showing little pleiotropy.

**Table 1:** Results of causal associations between age at first birth, age at menopause, age at menarche and ovarian cyst.

Exposure	Outcome	Method	No. of SNPs	Beta	SE	OR (95% CI)	*p*	Q stastistic	*p*- heterogeneity	*p*- intercept
Age at first birth	Ovarian cyst	Inverse variance weighted	51	-0.09	0.03	0.91 (0.86-0.96)	0.001854			
		MR Egger	51	-0.20	0.12	0.82 (0.64-1.04)	0.101932	65.64	0.056	0.362
		Weighted median	51	-0.09	0.04	0.92 (0.85-0.99)	0.032656			
		Weighted mode	51	-0.07	0.09	0.93 (0.77-1.12)	0.459661			
		MR Presso	51	-0.09	0.03	0.91 (0.86-0.97)	0.003064			0.057
Age at menopau se	Ovarian cyst	Inverse variance weighted	35	0.05	0.01	1.05 (1.03-1.08)	0.000008			
		MR Egger	35	0.07	0.03	1.08 (1.01-1.14)	0.021477	35.48	0.352	0.043
		Weighted median	35	0.06	0.02	1.06 (1.02-1.09)	0.000468			
		Weighted mode	35	0.06	0.02	1.06 (1.01-1.11)	0.022650			
		MR Presso	35	0.05	0.01	1.05 (1.03-1.08)	0.000086			0.396
Age at menarche	Ovarian cyst	Inverse variance weighted	6	-0.10	0.05	0.91 (0.82-0.99)	0.037648			
		MR Egger	6	-0.04	0.23	0.96 (0.61-1.52)	0.879696	4.19	0.381	0.798
		Weighted median	6	-0.07	0.06	0.93 (0.83-1.05)	0.252069			
		Weighted mode	6	-0.05	0.08	0.95 (0.82-1.10)	0.550588			
		MR Presso	6	-0.10	0.04	0.91 (0.83-0.99)	0.074255			0.578

**Figure 3:**
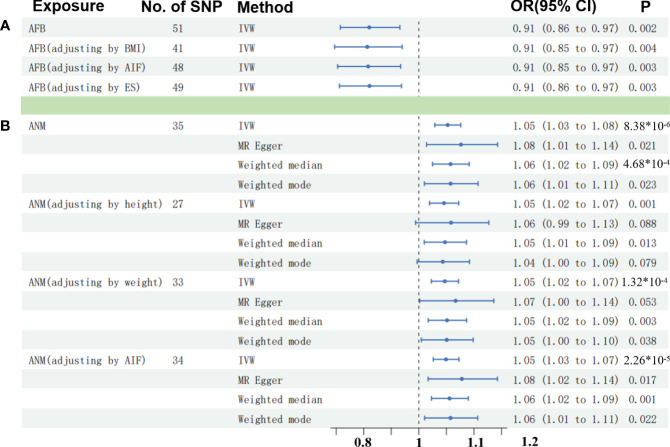
Comparisons of Mendelian randomization results. **(A)** Comparisons of Mendelian randomization results for AFB on OC. **(B)** Comparisons of Mendelian randomization results for ANM on OC. OC, ovarian cyst; AFB, age at first birth; ANM, age at natural menopause; BMI, body mass index; AIF, alcohol intake frequency; EK, ever smoked.

As modifiable risk factors of OC, obesity (BMI), alcohol intake frequency, and ever smoked may play a significant role in the etiology of OC. Adjusting the effect of confounders including BMI, alcohol intake frequency, and ever smoked, we also found the causal effect between AFB and OC (*p* < 0.005) (**Figure 3**; **Supplementary Table S4**). Moreover, we found that alcohol intake frequency and ever smoked played an unimportant role in the etiology of OC through the two-sample MR analysis. Meanwhile, the obesity with higher BMI increased the risk of OC (Beta: 0.106, *p* = 0.005) by our MR test (**Supplementary Table S4**).

### 3.2 Causal association of ANM on OC through two-sample MR

Similarly, we have found convincing evidence to support the causal relationship of genetically instrumented ANM with OC. As shown in **Table 1**, per-year increase in ANM was related to a 0.05 standard deviation risk increase of OC (OR = 1.05, 95% CI = 1.03–1.08, *p* = 8 × 10^−6^). Meanwhile, the detection results remained consistent using different methods (OR_per-SD increment in ANM_ [95% CI] was 1.08 [1.01–1.14] for MR-Egger regression, 1.06 [1.02–1.09] for weighted median, 1.06 [1.01–1.11] for weighted mode, and 1.05 [1.03–1.08] for MR-PRESSO). We did not identify any leverage points with a high influence in the leave-one-out sensitivity analysis (**Figure 2B**). Cochran’s *Q* statistic illustrated no heterogeneity among SNPs of OC. As a supplement to the MR-Egger analysis, the MR-PRESSO global test with a *p* of 0.396 and no outlier SNPs demonstrated no directional pleiotropy.

Then, an IVW-based multivariable MR (mvMR) was conducted to test the direct effect of ANM on OC accounting for the confounding effect from weight, height, and ever smoked. The results of mvMR remained consistent with our primary findings (*p* < 0.005) (**Figure 3**; **Supplementary Table S4**). In addition, to estimate the weight and height using mvMR, we found that genetically predicted weight showed little influence on the risk of OC (OR = 1.00, 95% CI = 1.00–1.01, *p* = 0.407), so did weight (OR = 1.03, 95% CI = 0.95–1.11, *p* = 0.495).

### 3.3 Causal association of AAM on OC through two-sample MR

Meanwhile, for genetically predicted AAM, we only observed significant association with OC using IVW (OR = 0.91, 95% CI = 0.82–0.99, *p* = 0.037648) (**Table 1**), while MR-Egger regression (OR_per-SD increment in AAM_ [95% CI], 0.96 [0.61–1.52]), weighted median approach (OR_per-SD increment in AAM_ [95% CI], 0.93 [0.83–1.05]), and weighted mode (OR_per-SD increment in AAM_ [95% CI], 0.95 [0.82–1.10]) remained non-significant. In addition, no heterogeneity and directional pleiotropy were found from the MR-Egger and MR-PRESSO test. Moreover, there are no confounding effect using the PhenoScanner method.

## 4 Discussion

In this work, we tested a putative causal relationship between three hormonal reproductive traits (AFB, ANM, and AAM) and OC influencing many women for the first time, making use of SNPs of strong IVs related to exposure (*F*-statistics: 921.51 for AFB, 1,148.44 for ANM, and 56.32 for AAM). We have found reliable evidence to demonstrate the causal effects of AFB, ANM, and AAM on OC using the two-sample MR analysis. Specifically, we genetically predicted that delayed AFB and AAM were associated with a decreased risk of OC, and similarly, we genetically predicted that younger ANM was related to a lower risk of OC. Therefore, a shorter reproductive period is associated with a lower risk of OC. After adjusting the effect of confounders including obesity (BMI), alcohol intake frequency, and ever smoked for AFB and weight, height, and ever smoked for ANM, a consistent causal effect was identified through the mvMR, proving the robustness of our findings.

When considering clinical significance, it is important to assess the magnitude of the effect and its practical implications in a real-world setting. While confidence intervals of approximately 1 indicate uncertainty, it does not necessarily mean that the results are not clinically significant. To determine clinical significance, it would be helpful to have more information on the OC, the large number of population being studied, and the causal determination of risk factors at the genetic level. Additionally, the interpretation of clinical significance may change depending on the field of study and the specific outcome being assessed.

Previous studies have found that there may be a relationship between AFB and ovarian cancer, and late AFB was associated with increased risk (26). Conversely, researchers summarized the AFB with risk of cancer and found that younger age (typically defined as 19 years or younger) at first birth is associated with an increased risk of cervical and endometrial cancers (27). Therefore, hormonal exposure of AFB has a strong correlation with ovarian disease due to certain reasons. First, reproductive factors such as AFB were very complicated and influenced more by environmental factors than by genetic factors. Second, estimates from previous epidemiologic studies are affected by confounders. For instance, smoking is one of the risk factors that have been considered for functional OCs, and a case–control study identified that the increase in BMI may reduce the adverse effect of smoking on the risk of functional OC (28). Thus, it is of vital importance to control the confounding factors in epidemiologic research. In our mvMR, we have controlled the effect of BMI, the alcohol intake frequency, and ever smoked, and we also obtained positive results after removing the confounders. Moreover, we tested the causal effect of confounders and outcome, showing a positive result for BMI with hundreds of IVs (**Supplementary Table S4**). Further investigations were needed to identify the causal relationship between BMI and OC.

As we all know, menopause was accompanied by the variation in hormone level, leading to many diseases in women. However, the biological mechanisms of hormonal factors on the influence of OC remain obscure. Recently, experimental research showed that the insufficient LH surge, intrafollicular changes in gonadotrophin receptors, and growth factors are the potential reasons leading to hormonally active OCs (4). During perimenopause, the hypothalamic arcuate nucleus and paraventricular nucleus are induced to pulse the secretion of gonadotropin-releasing hormone into the portal circulation due to the decrease in estrogen level, causing an increase in LH. Hence, older age of natural menopause may influence the LH level, thus leading to the elevated risk of OC.

As for the advantages of this study, an MR analysis was conducted to evaluate the causal effect between the reproductive factors and OC. Three distinct reproductive factors (AFB, ANM, and AAM) were incorporated to reflect the length of the reproductive period. The results clarified that prolonged exposure to estrogens as a consequence of a delayed menopause and early menarche increases the risk of the hormone-dependent disease of OC. Moreover, we performed a bidirectional two-sample MR analysis to avoid reverse causality, and the negative results were obtained as shown in **Supplementary Table S4**. Potential confounders such as BMI and ever smoked were also adjusted by mvMR, which makes the results more reliable and robust compared with observational studies.

As for the limitations of our study, at first, the number of our genetic instruments (SNPs) was less than 100, and further verification was needed to enhance the results. Second, participants of European ancestry were included in our MR analysis, which influenced our results’ external validity to other ancestry groups. Third, our research was performed using the overall OC rather than distinguishing disease subtypes including functional cysts, endometriotic cysts/blood cysts, dermoid cysts, serous cysts, and mucinous cysts. AFB and ANM may have a different effect on the various subsets of OC. Fourth, the MR-PRESSO GLOBAL test results of AFB showed minimal pleiotropy, while another effective method, the MR-Egger test, served as a supplement to show no directional pleiotropy (29). Furthermore, other social factors such as education and financial state may also confound our results without consideration of our MR analysis. Therefore, further studies should investigate the variety of subsets and adjust the social factors.

## 5 Conclusion

In summary, our findings demonstrated that reproductive factors (AAM, AFB, and ANM) play an important role in the risk of OC. Further research such as clinical trials and observational studies is needed to learn more about the relevant mechanisms.

## Data availability statement

The datasets presented in this study can be found in online repositories. The names of the repository/repositories and accession number(s) can be found in the article/**Supplementary Material**.

## Ethics statement

Ethical approval was not required for the study involving humans in accordance with the local legislation and institutional requirements. Written informed consent to participate in this study was not required from the participants or the participants’ legal guardians/next of kin in accordance with the national legislation and the institutional requirements.

## Author contributions

QS: Conceptualization, Data curation, Investigation, Software, Writing – original draft, Writing – review & editing. ZY: Formal Analysis, Methodology, Supervision, Validation, Data curation, Writing – review & editing.

## References

[1] Farghaly SA. Current diagnosis and management of ovarian cysts. Clin Exp Obstet Gynecol. (2014) 41:609–12. doi: 10.12891/ceog20322014 25551948

[2] Mobeen S Apostol R. Ovarian cyst. StatPearls (2023). Treasure Island (FL): StatPearls Publishing. 32809376

[3] Parazzini F La Vecchia C Franceschi S Negri E Cecchetti G. Risk factors for endometrioid, mucinous and serous benign ovarian cysts. Int J Epidemiol. (1989) 18:108–12. doi: 10.1093/ije/18.1.108 2722352

[4] Sasidharan JK Patra MK Singh LK Saxena AC De UK Singh V. Ovarian cysts in the bitch: an update. Top Companion Anim Med (2021) 43:100511. doi: 10.1016/j.tcam.2021.100511 33434678

[5] Morton A Teasdale S. Physiological changes in pregnancy and their influence on the endocrine investigation. Clin Endocrinol (Oxf). (2022) 96:3–11. doi: 10.1111/cen.14624 34724247

[6] Luo AJ Feng RH Wang XW Wang FZ. Older age at first birth is a risk factor for pancreatic cancer: a meta-analysis. Hepatobiliary Pancreat Dis Int (2016) 15:125–30. doi: 10.1016/s1499-3872(16)60063-2 27020627

[7] Yang HH Chen GC Zhou MG Xie LF Jin YY Chen HT. Association of age at first birth and risk of non-alcoholic fatty liver disease in women: evidence from the NHANES. Hepatol Int (2023) 17:303–12. doi: 10.1007/s12072-022-10429-1 36227515

[8] Li Z Gu M Cen Y. Age at first birth and melanoma risk: a meta-analysis. Int J Clin Exp Med (2014) 7:5201–9. PMC4307469 25664022

[9] Zhang X Huangfu Z Wang S. Review of mendelian randomization studies on age at natural menopause. Front Endocrinol (2023) 14:1234324. doi: 10.3389/fendo.2023.1234324 PMC10520463 37766689

[10] El Khoudary SR. Age at menopause onset and risk of cardiovascular disease around the world. Maturitas (2020) 141:33–8. doi: 10.1016/j.maturitas.2020.06.007 33036700

[11] Honigberg MC Zekavat SM Aragam K Finneran P Klarin D Bhatt DL. Association of premature natural and surgical menopause with incident cardiovascular disease. JAMA (2019) 322:2411–21. doi: 10.1001/jama.2019.19191 PMC7231649 31738818

[12] Magnus MC Borges MC Fraser A Lawlor DA. Identifying potential causal effects of age at menopause: a Mendelian randomization phenome-wide association study. Eur J Epidemiol. (2022) 37:971–82. doi: 10.1007/s10654-022-00903-3 PMC9529691 36057072

[13] Zhang N Liao Y Zhao H Chen T Jia F Yu Y. Polycystic ovary syndrome and 25-hydroxyvitamin D: A bidirectional two-sample Mendelian randomization study. Front Endocrinol (Lausanne). (2023) 14:1110341. doi: 10.3389/fendo.2023.1110341 36967791 PMC10034407

[14] Zhu J Niu Z Alfredsson L Klareskog L Padyukov L Jiang X. Age at menarche, age at natural menopause, and risk of rheumatoid arthritis - a Mendelian randomization study. Arthritis Res Ther (2021) 23:108. doi: 10.1186/s13075-021-02495-x 33836822 PMC8034136

[15] Mills MC Tropf FC Brazel DM van Zuydam N Vaez A eQTLGen Consortium. Identification of 371 genetic variants for age at first sex and birth linked to externalising behaviour. Nat Hum Behav (2021) 5:1717–30. doi: 10.1038/s41562-021-01135-3 PMC7612120 34211149

[16] Day FR Ruth KS Thompson DJ Lunetta KL Pervjakova N Chasman DI. Large-scale genomic analyses link reproductive aging to hypothalamic signaling, breast cancer susceptibility and BRCA1-mediated DNA repair. Nat Genet (2015) 47:1294–303. doi: 10.1038/ng.3412 PMC4661791 26414677

[17] Kurki MI Karjalainen J Palta P Sipilä TP Kristiansson K Donner KM. FinnGen provides genetic insights from a well-phenotyped isolated population. Nature (2023) 613:508–18. doi: 10.1038/s41586-022-05473-8 PMC9849126 36653562

[18] Hemani G Zheng J Elsworth B Wade KH Haberland V Baird D. The MR-Base platform supports systematic causal inference across the human phenome. Elife (2018) 7:e34408. doi: 10.7554/eLife.34408 29846171 PMC5976434

[19] Gill D Karhunen V Malik R Dichgans M Sofat N. Cardiometabolic traits mediating the effect of education on osteoarthritis risk: A mendelian randomization study. Osteoarthritis Cartilage. (2021) 29:365–71. doi: 10.1016/j.joca.2020.12.015 PMC7955282 33422704

[20] Yoshikawa M Asaba K. Educational attainment decreases the risk of COVID-19 severity in the European population: A two-sample mendelian randomization study. Front Public Health (2021) 9:673451. doi: 10.3389/fpubh.2021.673451 34150709 PMC8212884

[21] Huang W Xiao J Ji J Chen L. Association of lipid-lowering drugs with COVID-19 outcomes from a Mendelian randomization study. Elife (2021) 10:e73873. doi: 10.7554/eLife.73873 34866576 PMC8709572

[22] Burgess S Davey Smith G Davies NM Dudbridge F Gill D Glymour MM. Guidelines for performing Mendelian randomization investigations: update for summer 2023. Wellcome Open Res (2023) 4:186. doi: 10.12688/wellcomeopenres.15555.3 32760811 PMC7384151

[23] Verbanck M Chen CY Neale B Do R. Detection of widespread horizontal pleiotropy in causal relationships inferred from mendelian randomization between complex traits and diseases. Nat Genet (2018) 50:693–8. doi: 10.1038/s41588-018-0099-7 PMC6083837 29686387

[24] Bowden J Davey Smith G Burgess S. Mendelian randomization with invalid instruments: effect estimation and bias detection through egger regression. Int J Epidemiol. (2015) 44:512–25. doi: 10.1093/ije/dyv080 PMC4469799 26050253

[25] Yin KJ Huang JX Wang P Yang XK Tao SS Li HM. No genetic causal association between periodontitis and arthritis: A bidirectional two-sample mendelian randomization analysis. Front Immunol (2022) 13:808832. doi: 10.3389/fimmu.2022.808832 35154127 PMC8825874

[26] La Vecchia C Decarli A Franceschi S Regallo M Tognoni G. Age at first birth and the risk of epithelial ovarian cancer. J Natl Cancer Inst (1984) 73:663–6. 6590912

[27] Merrill RM Fugal S Novilla LB Raphael MC. Cancer risk associated with early and late maternal age at first birth. Gynecol Oncol (2005) 96:583–93. doi: 10.1016/j.ygyno.2004.11.038 15721398

[28] Holt VL Cushing-Haugen KL Daling JR. Risk of functional ovarian cyst: effects of smoking and marijuana use according to body mass index. Am J Epidemiol. (2005) 161:520–5. doi: 10.1093/aje/kwi080 15746468

[29] Burgess S Thompson SG. Interpreting findings from Mendelian randomization using the MR-Egger method. Eur J Epidemiol. (2017) 32:377–89. doi: 10.1007/s10654-017-0255-x PMC5506233 28527048

